# Supplementary material for: Reconciling Mining with the Conservation of Cave Biodiversity: A Quantitative Baseline to Help Establish Conservation Priorities
Source: PLoS One. 2016 Dec 20;11(12):e0168348. doi: 10.1371/journal.pone.0168348 (PMC5173368; doi:10.1371/journal.pone.0168348)
Supplement: S1 Dataset — (ZIP) [file pone.0168348.s002.zip › Taxa/Serra Norte/SN_2013/N5S/Lista N5S-23.pdf]

CAVIDADE N5S-0023

Classe	Ordem	Fam/Outros	Gên/Outros	Espécie	S-U	U-S	Zon.
Arachnida	Acari	(Oribatida)		sp.3	X		P
Arachnida	Acari	Eupodidae	*Linopodes*	sp.1		X	P
Arachnida	Amblypygi	Phrynidae	*Heterophrynus*	*longicornis*	2	1	P
Arachnida	Araneae	Corinnidae		jovem	1		P
Arachnida	Araneae	Ochyroceratidae		jovem	X	X	P
Arachnida	Araneae	Salticidae		jovem	X		P
Arachnida	Araneae	Theridiidae	*Theridion*	sp.3	X		P
Arachnida	Araneae	Theridiosomatidae		jovem		X	P
Diplopoda	Polydesmida	Chelodesmidae		sp.1	1		P
Insecta	Coleoptera	Carabidae	*Gen*.n.	sp.17		X	
Insecta	Diptera	Culicidae	*Culex*	sp.		X	P
Insecta	Diptera	Keroplatidae	*Euceroplatus*	sp.		X	P
Insecta	Diptera	Psychodidae	*Sciopemyia*	*sordellii*	X	X	P
Insecta	Diptera	Tipulidae	Tipulinae	sp.	X		P
Insecta	Heteroptera	Ceratocombidae		sp.		X	P
Insecta	Heteroptera	Lygaeidae	Rhyparochrominae	sp.		X	P
Insecta	Homoptera	Cixiidae		sp.1	X	X	P
Insecta	Hymenoptera	Diapriidae		sp.2		X	P
Insecta	Hymenoptera	Formicidae	*Acromyrmex*	sp.1		X	P
Insecta	Orthoptera	Phalangopsidae	*Phalangopsis*	sp.	2	12	P
Gastropoda	Pulmonata	Subulinidae	*Lamellaxis*	sp.	X		P
Malacostraca	Isopoda	Philosciidae		sp.2		X	P
